# Frequent premature ventricular complexes are benign!?

**DOI:** 10.1093/europace/euac263

**Published:** 2023-02-03

**Authors:** Rakesh Latchamsetty, Frank Bogun

**Affiliations:** Section of Electrophysiology, Division of Cardiovascular Medicine, Department of Internal Medicine, University of Michigan, 1500 E Medical Center Dr, Ann Arbor, MI 48104, USA; Section of Electrophysiology, Division of Cardiovascular Medicine, Department of Internal Medicine, University of Michigan, 1500 E Medical Center Dr, Ann Arbor, MI 48104, USA

**This editorial refers to ‘Prognostic implication of premature ventricular contractions in patients without structural heart disease’, by R. Scorza *et al*., https://doi.org/10.1093/europace/euac184.**

Our understanding of the prognosis of patients with frequent premature ventricular complexes (PVCs) has evolved considerably over the past 40 years. Although determined to be a marker of poor prognosis in patients with prior myocardial infarction,^1^ PVCs in patients with ‘apparently normal’ hearts were initially considered benign.^2^ This initial perception has changed following numerous studies showing frequent PVCs to not just be correlated with, but causative for cardiomyopathy with their elimination frequently resulting in improvement or normalization of cardiac function.^3^ While there is no exact cut-off in PVC burden that portends a high risk of cardiomyopathy and there are factors independent of PVC frequency that impact prognosis, it is recommended that a PVC burden of >10% warrants routine monitoring of cardiac function independent of patient symptoms, and intervention should be considered in patients that exhibit either limiting symptoms or deterioration in cardiac function.^4^ The prognosis in patients with lower PVC burdens with apparently normal hearts and whether PVCs play a causative role in worse outcomes remains an area of investigation.

In this edition of *Europace*, Scorza et al.^5^ compared the outcomes of a cohort of patients from three major hospitals in Stockholm with PVCs and without apparent heart disease to an age and sex matched control group from the general Swedish population. Patients in the PVC group had no previous significant cardiac history and were required to have a normal echocardiogram and exercise stress testing. The authors report that the mortality and cardiovascular morbidity over a period of 5.2 years were similar in the patients with PVCs compared with the control group. They conclude that patients with PVCs who receive a thorough cardiac evaluation ruling out structural heart disease should be given a reassuring message regarding their mid-term prognosis.

This study raises several interesting questions about our evaluation in patients with PVCs and our ability to establish their prognosis. Although the findings may seemingly contradict other studies that have shown even modest PVC burdens to be associated with worse long-term outcomes including the development of heart failure or even mortality, there are key study differences that can help reconcile these findings. In one of the most robust longitudinal studies evaluating outcomes in patients with PVCs and apparently normal hearts, Dukes et al.^6^ reported a decrease in cardiac function, an increase in heart failure, and an increase in mortality among patients with a higher frequency of PVCs. Interestingly, much of these differences were noted after 5 years of follow-up, suggesting the follow-up time in the current study may have been inadequate to detect these differences. Other studies have confirmed these findings including one where PVC symptoms >60 months was an independent predictor for the development of cardiomyopathy.^7^ By identifying patients with normal initial cardiac testing, it is likely that the current study pre-selects patients that are relatively early in their diagnosis of PVCs.

The term ‘apparently’ normal heart alludes to the fact that echocardiography and stress testing may be insufficient to completely rule out the presence of structural heart disease. Scarring as defined by cardiac magnetic resonance imaging (CMR) can be detected by late gadolinium enhanced areas in the myocardium that may be present even in patients with normal left ventricular function. About one-fourth of patients with frequent PVCs despite having no obvious heart disease have scarring by CMR and some of these patients have inducible ventricular tachycardia despite an ‘apparently’ normal heart. These patients are at risk for sudden cardiac death and would not have been identified if it were not for their frequent PVCs.^8^ Hence, PVCs can be markers of the presence of structural heart disease and echocardiography and stress testing in our opinion is insufficient to rule out structural heart disease. The presence of scarring is of key importance as patients with frequent PVCs and scarring associated with inducible ventricular tachycardia have an increased risk for cardiac mortality despite normal left ventricular function.^8^ Furthermore, scarring is an independent risk factor for PVC-induced cardiomyopathy that can increase cardiovascular morbidity.^9^ We also know that the probability of having scar in the absence of prior myocardial infarction increases with comorbidities including diabetes,^10^ atrial fibrillation,^11^ lower pre-existing ejection fraction, and age.^12^ In the present cohort, since patients with an impaired EF were excluded and the population is relatively young with a median age of 59, this selected a lower risk patient group for the presence of scarring.

Finally, we must take note of the limitations in the current study design that will limit the interpretability of the results, some of which the authors have recognized. The PVC group consisted of patients with not just baseline preserved cardiac function by echocardiogram, but also primarily patients with a functional class enabling the completion of an exercise stress test. Another important confounder, as the authors mention, are that the PVC group were from major medical centres in Stockholm and the control group may not have benefited from the same level of follow-up care. These differences in the two groups are quite significant and temper our interpretation of their similar outcomes presented in the current manuscript.

In conclusion, the current study does suggest that baseline healthy patients with PVCs and the ability to complete exercise stress and echo testing with favourable results and close clinical follow-up as would be expected at a secondary medical centre can follow a favourable short to mid-term course as a group. However, these findings should be balanced with caution that a subset of this group, who may be identified with further cardiac testing such as MRI, may be found to have a higher risk. Moreover, we would caution against simple reassurance and would continue to recommend close long-term follow-up in such patients.

## Data Availability

None declared.
